# ‘Just Let Them Play’: Complex Dynamics in Youth Sport, Why It Isn’t So Simple

**DOI:** 10.3389/fpsyg.2021.700750

**Published:** 2021-09-16

**Authors:** Christine Nash, Jamie Taylor

**Affiliations:** ^1^Institute for Sport, PE & Health Sciences, The University of Edinburgh, Edinburgh, United Kingdom; ^2^Grey Matters Performance Ltd., Stratford-upon-Avon, United Kingdom; ^3^School of Health and Human Performance, Faculty of Science and Health, Dublin City University, Dublin, Ireland

**Keywords:** role of the coach, expertise, long-term commitment, complexity, PJDM, criticality, coherence

## Abstract

This ethnographic case study examines the long-term impact of youth sport coaching within tennis, using observations, field notes, and interviews as data sources. We highlight the complexities that youth sport coaches face in their role in developing young players within, in this example, tennis, but suggest that these issues are transferable across the youth sport context. There are some key messages for youth sport coaches and sporting organisations arising from this study, particularly around the role of a youth sport coach. We advocate an expertise approach to developing youth sport coaches due to the many roles, within their sport and from a biopsychosocial perspective, that they have to navigate. Additionally, we suggest that simplistic narratives in youth sport coaching are misplaced.

## Introduction

Over the previous 15 years, numerous conceptual models of youth sport coaching have been suggested by researchers (Eime et al., 2013; Holt et al., 2017; Jones et al., 2017). These models have proposed a range of different aims for youth sport, such as the development of “competent, confident, connected, compassionate, character-rich members of society” (Fraser-Thomas et al., 2005, p. 33). In turn, there have been suggestions about the methods that coaches might deploy to achieve these desirable outcomes. For example, the need for an appropriate context for self-discovery, children being surrounded by positive external assets, acquiring internal assets, and benefiting from the findings of an on-going evaluation system (Petitpas et al., 2005). For the coach aiming to maximise the experience of their participants, many of these recommendations, such as the overall classification of environments as being positive or negative (e.g., Petitpas et al., 2005), seem to stand in contrast with literature in the broader domain which has placed significant emphasis on the “shades of grey” involved in the coaching process (Jones et al., 2013; Collins et al., 2015). In parallel, much of the coaching literature has long been criticised for being out of touch with the reality of practice, lacking recognition of the nuanced contextual challenges inherent in the coaching process (Cushion et al., 2006).

The biopsychosocial model is an interdisciplinary framework that views biological, psychological, and social factors as interacting features of human development (Engel, 1977). In the field of sport and exercise, the BPS model would suggest that the complexity of the coaching process appears to be the result of interactions between biological, psychological, and social factors (Bailey et al., 2010; Collins et al., 2012; North, 2017). We can therefore see participant development as an inherently individual process influenced by a range of complex and dynamic factors (Abbott et al., 2005; Van der Sluis et al., 2019). Psycho-socially, the differences between different coaching environments such as: participation, development, and elite sport are well established (Collins and MacNamara, 2017; Lyle and Cushion, 2017). Collins et al. (2012) suggested a “Three Worlds” model for participant development, the first being elite referenced excellence (ERE), where achievement is measured against others, with the ultimate goal of winning. The second, personally referenced excellence (PRE), where achievement is based on improving one’s best. The third being participation for personal well-being (PPW), either socially, personal fulfilment or self-concept. This model suggests that need for a continuum approach, recognising that the motivations of participants will shift over time and not necessarily just reflecting their level of performance (Collins et al., 2012).

Given the biopsychosocial complexity, it has been suggested that effective practice relies on the ability to make decisions at a number of levels (Abraham and Collins, 2011b; Lyle and Muir, 2020). At the macro level, decisions should work backward from long-term desired outcomes, taking account of long-term participant needs and the broader agenda that the coach works within (e.g., long-term participation or membership of the club). At the meso level or medium-term level, this long-term agenda should be taken into account, but influenced by particular issues (e.g., seasonal climate and participant lifestyle). At the micro level, the coach should look to nest their moment to moment and short-term coaching decisions within these longer-term agendas (e.g., motivational status of participant). Thus, to navigate the complexities of development, there is a need for a professional judgment and decision making (PJDM) approach, with the coach ideally weighing up the tentative balance of short- and long-term needs (Taylor and Collins, 2020). In turn, the coach needs to carefully consider the relative ranking of these needs, ideally working toward a long-term nested agenda (Abraham and Collins, 2011b).

Critical to effective decision making is an expertise-based focus in the development of coaches (Nash et al., 2012). An expertise approach would emphasise the need to develop a large base of declarative knowledge and apply this knowledge through the use of a variety of cognitive skills (Nash and Collins, 2006; Nash et al., 2012). Indeed, deep conceptual knowledge and an understanding of *why* something works appears to be the primary basis for flexibility, adaptability, and the ability to innovate (Hatano and Inagaki, 1986): “Adaptive expert’s knowledge representations are more flexible, allowing them to respond to novel situations more effectively” (Gube and Lajoie, 2020, p. 2). This flexibility appears to underpin the pedagogical agility of the coach and their ability to make decisions in a complex and changing environment (Mees et al., 2020).

Although expertise-based approaches have been prevalent in the coaching literature for a number of years, a range of pseudoscientific approaches based on the “right way to do it” are perpetuated in coach education and wider discourse (Bailey et al., 2018; Stoszkowski et al., 2020). This trend toward oversimplification has seen the widespread promotion of athlete centredness, autonomy-supportive coaching and athlete empowerment without critical consideration, or granular consideration of meaning (Alder, 2018).

As an example, there are a number of popular memes in lay coaching culture such as “let them play,” broadly conveying the idea that adult input interferes with the quality of experience for children in youth sport (Lynch, 2016; Cairney et al., 2018). Many similar ideas are prominent on social media, a growing source of information for the wider coaching population (Stoszkowski and Collins, 2016). This, despite some identified challenges with discovery learning as a methodology (Kirschner et al., 2006) and a wide body of research emphasising the need for coaches to utilise a range of different approaches in their coaching practice (Abraham and Collins, 2011a; Pill, 2020). Whilst some may suggest that the role of the youth sport coach is a relatively simple reproduction of a “best way,” this stands in contrast with a significant body of research and, perhaps, the reality of the coaching context (cf. Nash and Collins, 2006; Abraham et al., 2009).

An area of particular concern in talent development (TD) coaching has been the role of challenge (Collins and MacNamara, 2012) and the proposed need to develop psycho-behavioural skills prior to challenging experiences to promote further learning and development (Savage et al., 2017, 2021). Given the range of participant motivations in the youth sport context and the promotion of long-term engagement, there is a need for the coach to be pedagogically agile (Mees et al., 2020).

Yet, there appears a point of contention in the literature. For some, meeting the needs of PPW participants is a matter of maximising fun, emphasising short term engagement on a session-by-session basis, rather than holding a longer-term outlook (Trudel and Gilbert, 2006; Côté and Hancock, 2016). For others, there is a need for a longer-term outlook, systematically developing perceived and actual competence through skill development (MacNamara et al., 2015). In addition, with challenge being a recommended feature of youth sport (Fraser-Thomas and Côté, 2009) and the role of challenge for ERE and PRE participants becoming better understood (Collins et al., 2016b; John et al., 2019). This is confusingly contradicted by some advice in youth sport coaching, for example: “sport and physical activity offer youth opportunities to experience challenge…while…decreasing their stress” (Fraser-Thomas et al., 2005, p. 24).

In addition to these apparent disagreements within the literature and obvious difficulty of managing the challenge load of participants, the coach also needs to take account of the number of different sources of information available to the athlete, such as parents and peers (Harwood and Knight, 2015). All of which have the potential to significantly mediate the coaching process (Pankhurst et al., 2013). This is both a result of the number of different perspectives offered to young people, but also their individual biopsychosocially mediated response to their experiences and the input they receive (Taylor and Collins, 2021). The need for coherent messaging is a well-established feature of effective TD practice (Martindale et al., 2005; Henriksen and Stambulova, 2017), yet is problematic for the majority of coaching contexts (Webb et al., 2016). Thus, whilst the coaching context might seek to offer a coherent experience and encourage the navigation of a variety of challenges, it relies on the input of stakeholders external to the coaching environment (Bjørndal and Ronglan, 2018).

Given the weight of this biopsychosocial complexity, it appears unclear how desirable outcomes in the youth sport context can be attained if coaches operate on a sessional basis using simple guidelines. It is likely that coaching PPW participants presents just as great a demand on a coach’s PJDM as any other context. Yet, the dilemmas of practice in youth sport and how the coach balances a range of short and long-term objectives are poorly understood (Jones and Wallace, 2006; Abraham and Collins, 2011b).

Therefore, on a broader longitudinal basis, we set out to understand, longitudinally and in depth, the nature of the coaching milieu and the types of dilemmas faced by the coach in meeting the needs of PPW participants. Similarly, given the overall emphasis on supporting long-term participation, there is a significant gap in knowledge for the coach seeking to promote healthy outcomes for participants (Côté and Hancock, 2016).

## Materials and Methods

This article is concerned with reporting and discussing the results from a single-site ethnographic study that was conducted over a period of 42 months (March–September each year) of a 6-year period. According to Yin (2009) a case study approach allows the researcher to explore participants through complex interventions, relationships, communities, and programmes. Baxter and Jack (2008) comment further, that a rigorous qualitative case study offers opportunities to explore or describe a phenomenon in context, using a variety of data sources. This case study of coaching within a junior tennis section fits well with ethnography whose primary purpose is to capture the routine and everyday activities and to understand the meaning from the participants’ viewpoint, in this case tennis coaches and players (O’Reilly, 2012). Ethnography investigates below surface appearances, seeking to expose social practices in complex and dynamic situations, such as youth sport coaching (McKenzie et al., 2005). Moreover, the variety of data collection methods associated with ethnography (e.g., prolonged fieldwork, observation, and informal interviews) can illuminate the complexity of psychosocial processes in groups, especially within coaching (cf. Rock, 2001). To be successful, ethnography involves regular in-depth contact over a prolonged period of time, in this case 42 months of weekly contact at Hill View Tennis Club (HVTC). Initially, a presentation was given to the families, tennis players and coaches to explain the details of the study. After that, an informed consent was given to each family to read, indicating the background and commitments of being part of the study. Institutional ethical approval was granted prior to the study and informed consent was completed by both parents and tennis players. In line with Mannay and Morgan (2015) we were utilising a holistic ethnographic approach but have described individual elements of the data collection site and process here.

### Hill View Tennis Club

Hill View Tennis Club (pseudonym) is located in a small town in the commuter belt in Central Scotland with a prevalence of higher income and private housing. The tennis club has a small number of adult players but a thriving junior section, encouraged by regular tennis coaching throughout the tennis season (March–September). The club does not operate formally from October to February but members are still able to access the courts. HVTC encourages youth participation by offering free membership for those signing up to the coaching sessions. The coaching sessions were held twice a week and play was encouraged outwith these times to consolidate and develop skills. The definition of junior at the club was anyone under 18 years of age, however, the majority of participants attending the coaching regularly were 10–15 years of age. There was also an introductory group that worked with young people who were new to tennis, sampling the sport, and after they had completed this set of lessons, they tended to progress to the coaching sessions that are the focus of this study. The move to these coaching sessions demonstrated for most of the young people, a commitment to tennis and the start of the specialising phase, although in the initial stages, many were still involved in other sports and recreational activities. During this study there were more females involved in the tennis coaching than males (62% female on average) and, the females reported practising more between coaching sessions, generally with friends or parents.

### Field Notes

The first author of this article carried out observations of weekly coaching sessions, coaches and participants and interviewed young players, parents and coaches. Field notes containing the results of observations were kept and were written up when she was not in situ. Field notes consisted of oral and written records of incidents, events, documents, and unusual occurrences. They included electronic diary entries, oral records using a digital voice recorder, and handwritten notes.

### Informal Interviews

The first author carried out small group interviews (3–5 people in each group) with 38 young people between the ages of 12 and 16 years. These informal interviews were conducted courtside at HVTC at the start or completion of training sessions. These people had all been involved in the observation phase at varying points over the course of the study and some (21) were interviewed on more than one occasion. These interviews happened three times per year and lasted 20–30 min and were ad hoc, dependent upon the availability of players. The participants were asked what other sports/activities they were involved in, how interested they were in tennis and continuing playing and attending coaching sessions, how much they enjoyed competition and how the coach influenced their ongoing participation and commitment to tennis.

Informal interviews with three coaches were also held at the start and end of the coaching sessions each year, giving a total of 12 interviews. Each coach was responsible for the coaching sessions for two seasons and there were two female coaches and one male coach. The coaches were asked about their philosophy and approach to coaching tennis, their perceptions of their role as a coach and their thoughts on player development. Eleven parents were also interviewed informally when they arrived to collect their children from the coaching sessions. These interviews lasted 20–30 min and were again ad hoc, dependent upon who was available. Care was taken to speak to parents individually and only on one occasion each. The parents were asked about the other sport/activities their child participated in and to assess the level of commitment they displayed to each, as well as their views on the role and effectiveness of the tennis coach.

The interview questions were based on the interview guide from a previous study (Nash et al., 2008), that examined how philosophy and beliefs impacted on the perceived role of the coach. This ethnographic case study is focusing on coaches in youth sport, tasked with developing young tennis players but also considering the views of the tennis players and their parents, key to this age and stage of participation.

### Data Analysis

Hammersley (2006) emphasised how crucial the tension between participant and analytic perspectives when analysing ethnographic data, this tension arising from, on one hand, the perspective of the group being studied, in this case, the coaching environment in junior tennis versus the “making sense” of analysis perspective. Hammersley (2018) continues to highlight that ethnographic research entails a longer-term data collection process in natural environments that relies on collecting a range of types of data but based on observation. As a result of the holistic nature of ethnography, data analysis can be complicated. According to Gibbs (2007) there is no single method accepted by all ethnographic researchers that can serve as a strategy for the analysis of data collected in the field.

In this case all data analysis consisted of an iterative process of theme building (Fetterman, 2010). We identified text segments from field notes and interviews category labels to the segments and sorted all text segments that related to an area or subsequently a theme. We used the constant comparative method of analysing the data (Glaser and Strauss, 1967; Lincoln and Guba, 1985), manually reviewing the data repeatedly and coding looking for similarities, differences, groupings, patterns, and items of particular significance (Mason, 1996).

### Methodological Integrity

There is considerable debate around the concepts of reliability and validity and attaining rigor in qualitative research. In this study we investigated the experiences of all participants over 42 months to attempt to theorize inductively and reach insights that can be interpreted and applied to youth sport environments and their coaches. In line with Burke (2016) and Bradshaw et al. (2020), we applied four quality “markers” to this study:

1. This was a study that took place over six seasons, signifying prolonged immersion within the club environment – a key component of credibility.
2. There were a number of data sources accessed – observations, interviews, focus groups – that demonstrate the wide-ranging nature and rigour of the presented data.
3. Critical friends were consulted through the data collection and analysis phases resulting in sincerity of findings.
4. Each theme is presented using rich, contextualised portrayals so that data is easily transferable to other youth sport settings, achieving resonance.

Smith and McGannon (2018) examine the concept of universal criteria, the belief that a predetermined list of criteria can be used to judge qualitative research can be applied to any form of inquiry regardless of its intents and purposes “when a preordained and fixed quality appraisal “checklist” is used, research risks become stagnant, insipid, and reduced to a technical exercise” (p. 115). We adopt a pragmatic approach to research, and as such, consider this study to be methodologically coherent, given the philosophical assumptions underpinning this research are the most appropriate methods in the circumstances (Mayan, 2009).

The first author was a member of HVTC, played regularly and helped out as an assistant coach at the coaching sessions so was a familiar figure around the courts and clubhouse. This level of access is considered to be insider research, bringing benefits such as deeper knowledge and interaction (Greene, 2014), however, we employed reflexivity as a strategy in this ethnographic case study to enhance the rigour of the research (Berger, 2013). Reflexivity as a process is introspection on the role of subjectivity in the research process. A reflexive logbook was used to record personal thoughts, reactions, and questions about encounters and interactions with participants. The process of completing this logbook helped to stimulate self-awareness, criticality, and flexible thinking during data collection (Palaganas et al., 2017). The latter particularly important given the first author’s role as a member of the club as this process influences not only the research participants but the researchers themselves.

## Results

The results derived from this longitudinal ethnographic fieldwork demonstrated the importance of the role of the coach within the TD process in youth tennis participation. The three key themes arising from the data analysis were:

• The importance of challenge.
• The difficulty of offering appropriate support.
• Working with biopsychosocial dynamics.

The participants’ own voices are used to portray events, and pseudonyms are used to ensure confidentiality. Each theme is exemplified by two vignettes that were developed from field notes, observations and interactions, as suggested by Jarzabkowski and Kaplan (2014) and Michaud (2014) as a method of illuminating a particular event in ethnographic research. These themes are reinforced by the interview and observation data collected from parents and coaches.

### The Importance of Challenge

Part of the role of every coach should be to challenge their athletes to develop and this has the added benefit of motivation during practice, provided the coach has the necessary knowledge and experience to plan and adapt to changing circumstances during coaching sessions (Nash et al., 2011).

In this instance, the coach’s deliberate use of challenge for a “comfortable” player yielded significant performance improvements and over the longer term, resulting in a greater engagement with tennis. It appeared that this deliberate coach intervention was critical to challenge Robert, who appeared to be getting bored and frustrated with tennis. It is also notable, that initially, the coach helped him work through a deliberately *unenjoyable* process, one that he had not experienced before and required him to deploy a range of unfamiliar skills to navigate.

More broadly, this pointed to one of the dilemmas faced by all coaches for all players, the translation of performance in training or practice into the competitive environment. In general, the players tended to view competition as an outcome and therefore place more importance on results, an aspect that was often highlighted by parents as well. For example, a mother asked the question “why does Jake spend so much time at coaching and hardly ever get to play in matches. He doesn’t even get to play many matches at the club-it’s all about the strokes.” This was reinforced by another parent (mother) reporting: “Susan says that all the practice is boring, and they don’t play enough competitions so they can see who is best.” However, another parent disagreed, saying “My daughter does not enjoy the competition and would rather just practice.” The mixed views of parents and perceptions of players appeared to add to the difficulty faced by the coach in using appropriate challenge and meet the needs of individuals.

Similarly, the coaches expressed differing views with Mike (coach), initially not considering the use of challenge beyond formal competitive settings. Even after gaining more experience coaching within the club, he viewed his role within practice as “preparing players for competition using a number of different methods, but mainly *I just let them play*.” When pushed, he still thought success in competition was what defined his success as a coach. Amy however, said, “I know that we need to make things difficult in practice – push the boundaries but I find it very difficult to do this consistently. Some of the players enjoy the competitions but others get nervous, over-excited so for me it is a challenge too. How do I encourage them to develop but not make them too stressed about matches?”

As Vignette 2 highlights when athletes are bored with practice, it can be simply because they do not feel challenged enough. As a clear parallel with Vignette 1, when some of the players did not feel challenged it seemed to lead to less-than-optimal concentration, often causing ineffective or disrupted practice. Notably, there were a variety of views expressed by parents in relation to the behaviour of players, with Alan’s father stating, “I really don’t see why the coach can’t just insist that everyone does what he says – it was very easy in my days – coaches shouted and players obeyed.” However, many of the other parents disagreed with Alan’s father saying, “the coaches have a difficult job dealing with bad behaviour with a few players and that spoils the coaching for all the players who want to pay attention” and “why can’t the coaches just chuck the troublemakers out of the session – that would solve a lot of problems.”

**Vignette 1.**

The following excerpt highlights the difficulties experienced by the youth sport coach in periodising challenge within coaching sessions but emphasises the potential long-term benefits.

Some of the young players were playing in competitions, usually at the weekend, at a different time from the coaching sessions. Robert, one of the best players in the coaching sessions and the club, regularly found that he did not have much competition when taking part in coaching or more broadly, playing with other junior players. In short, he was a big fish in a small pond. When he went to competitions he often struggled, generally losing in the early stages. Fiona, the coach, realised that he was not being pushed during the coaching sessions so made two changes to try and stretch Robert. First, when she was able, she rallied with Robert and second, she arranged for Robert to play against two opponents. Over a period of weeks Robert was very uncomfortable as he was put under pressure and stretched, a situation he was not used to and did not enjoy. This appeared to test a variety of psycho-behavioural skills that he hadn’t previously needed to deploy in a tennis context. The increased range and level of competition required him to focus more during matches and deploy a range of metacognitive skills to outwit his opponents. It was only after a number of sessions, approximately 2 months, that Robert began to experience changes in his performance. It was only in hindsight that he appreciated the opportunity to raise his level of play.

**Vignette 2.**

The following excerpt demonstrates the need for careful planning to cater for individualised challenge within the youth coaching context.

Andrew had been in lessons for 3 years and was tall for his age as well as being well-coordinated from participating in a number of sports. During the coaching he picked up new skills very quickly but did not seem too interested in practising to consolidate them, in fact he appeared to be intent on disrupting practices rather than engaging with them. His coach, Mike, utilised a number of strategies to change Andrew’s highly disruptive behaviour, for example, excluding him, shouting at him and ignoring him but none of these methods worked. Over a period of months some of the other players started to copy Andrew, leading to some breakdowns in the coaching sessions. As a relatively inexperienced coach, Mike sought advice about how to deal with this worsening situation. He was advised that these difficulties may be avoided if he provided tasks that increased the tempo and concentration required. He needed to differentiate in his planning and recognise when he needed to adapt an activity to provide more challenge for individuals within his coaching sessions, particularly Andrew. Over a period of time, Mike worked hard to plan how to adapt his practices, developing options to implement when he recognises that attention is flagging. He expressed annoyance that he had not been able to see this earlier in his coaching journey as he had not realised the importance of adaptive planning or individual challenge previously.

Mike agreed with these parents’ point of view, saying “from my perspective, it would make my job a lot easier if I did not have to deal with those players who don’t want to listen.” Amy considered that players come to coaching for a variety of reasons, saying “sometimes it feels like we are just a cheap babysitting service but actually it gives us the opportunity to really motivate young people to take part in tennis – a game I love. As we get to know these players, we know what makes them tick, this is how we can make each session relevant and challenging for each of them.” Fiona reinforced this view saying “Kids come here because they are interested in tennis – we don’t force them so if we start getting behaviour problems then I think we need to start looking at what we are providing first. Young people are more complicated and expect more – there are so many options available to them now.” This raised an additional dilemma for the coach, if there were disruptive children in the playing group, this impacted on every participant’s experience. Mike often felt that he was having to spend a disproportionate amount of time catering to a small number of players, fitting group sessions to their needs. This had a direct impact on the amount of individual attention he could offer to other children.

### The Difficulty of Offering Appropriate Support

Being supportive is one of the key roles of a coach according to much published research (Cheon et al., 2015; Stebbings et al., 2016; Balk et al., 2019) however, given the demands of youth sport coaching, this was a persistently complex challenge for all coaches at the club. As the above vignettes suggest, challenge is a critical feature of PPW coaching and the interaction with support appears more a matter of participant perception, than balancing what a coach offers.

**Vignette 3.**

The following excerpt exemplifies the difficulties in dealing with a range of abilities within a youth coaching session.

It was the first week of tennis coaching and there were some new participants so Mike, the coach, said it was important to make them feel welcome and included within the group who mostly knew one another. When James appeared at the courts he was dressed in an outfit that was reminiscent of one of the Musketeers, the four Frenchmen who played in the 1920s – long white trousers, long sleeved white shirt and a blazer, reminiscent of Federer in 2009 and to top it all off he had a wooden racket. The other players took a look at James and then started sniggering – Mike did try to stop this but he was having a difficult time stopping laughing too. It did not appear to phase James at all as he appeared to be relishing the opportunity to take part in the coaching session. Unfortunately, he was not particularly coordinated and the weight of the wooden racket did not help so none of the other participants wanted to include him within their groups. This was a real problem for Mike, who wanted to offer his support but struggled to include him because he was so far behind the rest of the group. This problem persisted for 3 weeks, with Mike feeling that he was unable to cater for James’ needs as part of a group where he was the lowest ability. Unfortunately, after 3 weeks James did not return to the coaching.

Parents of these young tennis players also expected the coaches to be supportive and gave examples of the types of support that they valued from the tennis coaches. One father suggested that he liked “the way the coach encourages all of the players at different times during the session – it helps them to keep coming back.” A mother reinforced this point of view saying, “each of the players needs some different input – Michelle (her daughter) needs to be reassured that she is doing the shots right, she is very nervous about getting things wrong in the group but they are not all like that.” Another mother highlighted the support offered in non-tennis related situations similar to Vignette 4, saying “Grace uses the coach as a kind of sounding board – you know talking about things that bother her. She plays in a football team too and there are always all sorts of fall-outs and cliques happening there – I’m glad I don’t have to deal with that here too.”

**Vignette 4.**

The following excerpt shows the methods of support utilised by the coach to enhance engagement and motivation.

Jenny had been attending the coaching sessions for 3 years but never really seemed particularly committed to the coaching or practising between coaching sessions. Amy, the coach, had noticed that Jenny was not particularly engaged and made the decision to encourage Jenny over a number of weeks. This encouragement took the form of highlighting her competence in certain shots, being supportive of any effort and selecting her for some demonstrations. Midway through the coaching, approximately 3 months later, it seemed that a lightbulb had suddenly been switched on in Jenny and she became animated, chatty and enthusiastic about tennis. Jenny’s mother spoke to Amy after the coaching session to tell the difference that she had noticed in Jenny – more confident, motivated and interested. Jenny’s mother reported that Jenny had confided to her that the support and encouragement she had received from Amy had made an impact. Jenny continued to thrive during the coaching sessions and became the Junior Champion that year.

This vignette highlights the role that perceived competence can play in motivating participation and in the process of learning. It is also notable that in contrast to earlier vignettes, Amy deliberately utilised a very different approach to coaching Jenny, despite the apparent similarity in the need for greater engagement. In this example, the coach, Amy, chose to deliberately promote perceived competence and a positive emotional experience.

In discussion with Amy, she suggested that although she was deliberately shaping Jenny’s emotional experience, she believed that her ability to do this was shaped through the support of young people outside of the sporting context, such as helping at summer camps, saying: “I think they were just part of what I had to do and not really connected to sport at all.” At the beginning, I made mistakes from not reading the situation right, but I got better and listening really helped. This transferable nature of the support skills was also mentioned by Fiona, stating “I don’t really know how I do this – it’s an important part of the role but it’s not something that I have necessarily learned through tennis – more like life!” Both of these coaches were unaware that they were utilising empathic accuracy within their coaching role.

### Working With Biopsychosocial Dynamics

Participant development in sport is dynamic and non-linear and there are multiple pathways that individuals may take as they progress in their activity, so the youth sport coach requires an understanding of the various options. The interaction between biological, psychological and social factors are recognised as characterising human development and can be of particular relevance to youth sport coaches (Thiel and Munz, 2018). However, given the complexity of human factors, this can be problematic even to experienced youth sport coaches given the myriad of potential dilemmas and options.

Emotion in sport is an often neglected area, however, regulating feelings is a key element of successful practice, especially at high levels of performance (Jones et al., 2016). Amy’s experiences here highlight the reality, and indeed, the difficulties of coaching participants through adolescence during a time of emotional state in the learning process. Indeed, the emotional difficulties faced by Susan were a real challenge for Amy, the coach, offering support to Susan while going through adolescence, a period of significant biopsychosocial change (De Sousa Ferreira dos Santos et al., 2018). This particular instance was a real challenge for the coach, whilst offering support to Susan, she had to manage the disruption to the training session with 11 other players on court.

**Vignette 5.**

The following excerpt highlights the psycho-emotional backdrop of coaching that falls within the remit of a youth sport coach.

Amy, the coach, had set up a drill involving volleying from a feed into the corners of the court with 12 players. All of them were coping well with the practice, except for Susan who had missed five volleys in a row. Amy, understanding the benefit of challenge in practice, took the opportunity to stop Susan practising and take her aside to ask some questions. During the conversation, Susan started to cry and after much prompting explained to Amy that she was having a hard time at school and at home, and this was upsetting her so much that she really felt she could not concentrate on her tennis. Amy took the opportunity to discuss some options with Susan after coaching finished to help her with the ongoing situation. Amy gave Susan some advice about using the physical elements of practice to relieve stress, hitting the ball as a release. Amy encouraged Susan to use tennis as a way of clearing her mind, her personal “happy” space. She also made time to speak to Susan’s mother, with Susan’s permission, when she came to pick her up from coaching.

It was notable that Amy recognised her role as the coach both as needing to help children develop their skills and offer opportunities to manage their own emotions allowing them to cope with difficult situations in constructive ways. Amy considered that these psycho-behavioural skills as being critical to adaptive social interactions, saying “players at this age and stage are not very good at separating their feelings from one situation to another. They can come from school to coaching angry, motivated, hyper or upset depending on what they had last. We need to get them to regulate their moods and that’s what I was trying to do with Susan.” This skill-based approach emphasised that her coaching practice was about more than just tennis and that she believed her role was to equip players with a range of skills beyond the court. This approach was about far more than the short-term engagement on a session-by-session basis, instead, she sought to systematically develop psycho-behavioural skills over the long-term. Amy also believed that the coaching process was bi-directional in nature. That is, players needed an appropriate skillset to optimally engage with the coaching process, rather than relying on the coach, or the shaping of the environment alone. This deliberate shaping of biopsychosocial dynamics to support the longer term and broader development of participants can be contrasted with other coaches. For example, Mike’s perspective was that the easiest way to manage emotions was to keep the players occupied: “if I can keep them running around, it doesn’t really matter what they are doing, but keep them moving then they don’t have time to think.”

In this instance, Fiona appeared to doubt the effectiveness of a “let them play” approach. For Gary, playing against similar aged peers who were able to overpower him emphasised perceptions of his relative physical inadequacy and the psycho-social consequences of this. Highlighting the complexity of the challenges presented to the coach, Fiona was well aware of the non-linearity of player development and was able to understand Gary’s needs. Yet, being able to cater for these was highly challenging and again, required a long-term outlook.

Fiona, an experienced coach, articulated a number of nuances in the coaching process, valuing the need for planning and a long-term agenda, but also recognising the need to make quick decisions during sessions based on biopsychosocial dynamics (cf. Collins et al., 2016a): “I’ve never delivered a session exactly the way I planned it. I actually think if any coach does that then they are not paying attention to what is happening in front of them. The kids are not robots and they all have their differences, peculiarities, strengths and weaknesses so you have to tailor each activity to them.”

For Gary, there were few options for his ongoing enjoyment of tennis, because of his relative development. In this sense, there was an overall need for him to delay gratification and Fiona’s deliberate “selling” of a long-term vision of his development, this encouraged him to commit to participating in the sport, despite a lack of short-term fun.

## Discussion

The data from this long-term ethnographic case study highlighted three themes in relation to the dilemmas of practice inherent to the role of the coach in the youth sport setting: the importance of challenge; the difficulty of offering appropriate support and working with biopsychosocial dynamics. Perhaps as a result of the embedded and longitudinal nature of the investigation, our results demonstrate that despite previous characterisation as otherwise, there is a need to view every level of coaching practice as being a long-term endeavour. Given the size of the overall data set, we have selected a number of appropriate vignettes that illustrate the types of dilemmas that were persistently faced by coaches.

### Pedagogical Agility

All vignettes clearly presented coaches with a series of significant challenges to the coach’s PJDM. Indeed, all of these “problems” present the coach with decisions that have the potential to limit the participant’s long-term engagement in tennis and potentially physical activity as a whole. It appeared that the coaches’ ability to frame the problem was critical to their subsequent adaptive action, as attested to by the theories of Schon and Vygotsky in the sport coaching context (Nash, 2016; Jones et al., 2018). Similarly, for coaches to offer a genuinely developmental experience, they needed to draw upon a range of theories and a depth of understanding across human development. In some cases, they were unable to apply these concepts (for example, Vignette 4) and this led to participant drop out. Indeed, for one of the coaches, who was unable to draw on a range of different approaches, seeing coaching practice in simple terms (e.g., “let them play”) this appeared to be a significant factor in participants dropping out. This complex picture supports work suggesting that dropout from sport is a multi-layered and complex problem, with perceptions of competence a critical factor (Battaglia et al., 2021).

There was a difference in levels of pedagogical ability, and even knowledge, of these three coaches, Amy, Fiona and Mike. Both Amy and Fiona displayed a more sophisticated model, employing more adaptability in their PJDM across the vignettes, taking into account the player and the circumstances before taking action (Collins and Collins, 2017). Mike however, tended to use a “one size fits all” approach and was unable to make sense of the level of complexity inherent in his day-to-day interactions (Grecic and Collins, 2013). This led him to view coaching as being a relatively simple affair, at times being a matter of coach or environment input and player reception. This was markedly different to the perspectives of Amy and Fiona, who were better able to cater to the needs of those players that sat outside of the norm. In short, there was no universally appropriate solution that could meet the needs of every player at the club, all of the time (Bailey et al., 2010).

### The Nuances of Challenge and Support

There were clearly differences between players’, parents’ and coaches’ ideas around challenge and support as evidenced by the vignettes, observations and interviews. Challenge was interpreted by most as competition, the winning or losing of a tennis match in this case, however, challenge can also be putting a player in an uncomfortable position to encourage development and growth (Vignette 1) or to individualise coaching to meet the needs of all players in a session (Vignette 2). Perhaps there is a need for a wider conceptualisation of the potential utility of challenge, or indeed the deliberate use of stress to promote development and long-term engagement. In contrast to previous literature, the data clearly supports the notion that stress is not inherently a bad thing (cf. Fraser-Thomas et al., 2005). Coaches should consider reinforcing the notion of challenge within the coaching sessions and communicate to both players and parents that it can be more important than competitions (Santos et al., 2019).

Coaches regularly provide support to athletes, however, research suggests that they are not necessarily equipped with the skills to do so proficiently (Poucher et al., 2020). Young people who do not conform to norms, such as James in Vignette 3, are problematic to integrate into groups, especially when they are the newcomer. In these situations, the coach has to be clear on their role and be pedagogically flexible enough to meet the needs of all participants. For James, Mike’s play-based approach could not cater for his individual needs and this, along with a lack of appropriate support was a significant factor in him dropping out of the game.

Recognition of the emotional impact that sport can have on adolescents, and subsequently how to shape participant experience appears critical for youth sport coaches. Notably, the coach’s deliberate influence of the participant’s emotional state is missing from coach education and from the extant body of literature, which suggests the need for a universally “positive experience” (Côté and Hancock, 2016).

### The Expertise Approach

Given the overall nature and volume of dilemmas presented to the coaches, we would suggest that it is wholly inappropriate to suggest that coaching at youth sport level is a simple matter, or one that can be adequately done with basic levels of knowledge or skill. Similarly, the use of a competence-based approach to developing or assessing coaches would not seem to be meeting the needs of this group of coaches (Collins et al., 2015).

Learning is not a linear process with Vignette 6 highlighting there can be a number of biopsychosocial factors that contribute to progressions or regressions. Coaches need to be aware to the subtleties of each situation and react accordingly. Vinson et al. (2016, p. 55) consider “learning is seen as a process of interpretation and adaptation; thus problem solving and critical thinking are key components of effective learning environments”. The solutions that coaches need to find within their practice are therefore not all related to tennis. In fact, the vignettes, observations and interviews highlight that the most challenging and indeed most important dilemmas faced by coaches were pedagogical, often requiring significant empathic accuracy (Lorimer, 2013). In all of these situations, there appeared no straight forward answer. For example, how emotional tone influences the individual perceptions of the athlete and the social interactions that constitute the coaching process.

**Vignette 6.**

The following vignette is an example of the biopsychosocial dynamics at play and how they influenced the experience of all participants and the importance of the youth sport coaches’ understanding of the non-linearity in development.

Gary was an average player when he started the coaching sessions, he tried hard and consistently acted on the input of coaches. After approximately 2 years attending tennis coaching, Gary was becoming more competent in his play, but had not physically matured as much as most of his peers. Gary was not able to generate the same power as his peers, especially in the serve, due to his lack of height and musculature. His ability to cover the court was also compromised due to his lack of reach. As a result, he struggled with his self-esteem, especially as Gary compared himself with the more physically mature tennis players in his coaching group. His coach, Fiona, recognised that he was developing well as a youth tennis player but lacked the self-confidence and appreciation of his own self-worth within the group. She considered him to be a slow developer in comparison with some of his more physically mature peers, however, his attitude to hard work and commitment to practice emphasised his potential. Fiona did not want to put any extra pressure on Gary by highlighting his ability at an early age (12 years) but made the effort to encourage him at every opportunity to develop his sense of self-esteem as well as his tennis skills. She hoped that her restrained approach would allow Gary the time and space to develop the physical, psychological and social skills to complement his engagement with tennis. Gary continued to work hard and was heavily involved in coaching sessions but physically had not yet caught up with his peers.

Simple contexts are characterised by stability and clear cause-and-effect relationships that are easily recognisable, however, as we have shown in our data these dilemmas faced by coaches are multi-facetted. The complex decisions that coaches in youth sport are required to make in everyday practice have no obviously correct outcome, instead there are multiple permutations that may work. Each domain of expertise may have distinctive phenomena related to the field, however, practice and pattern recognition are keys indicators of expertise (Connors et al., 2011). All these vignettes highlight the need for coaches to develop PJDM to enable appropriate decisions to be made.

How coaches operationalise their PJDM, or display their expertise in action, is key, given the dynamic nature of sport coaching. In order for youth sport coaches to become expert problem solvers they require to be familiar with the context, not the sport-specific skills *per se*, but the knowledge of the individual/s and all aspects of the sport setting (Nash et al., 2012). This necessitates an expertise approach for coach development, such as that implemented in some medical professions, entailing strong personal relationships, the ability to manage complexity and “thinking on your feet” (Dickson et al., 2018 p. 459).

## Applied Implications

Building on the previous section and taking account of the need for research that offers implications for practice, we offer here five implications from the findings of this study that may be of use to youth sport coaches, coach developers and sporting organisations.

### Coaching as a Team Game – the Need for Coherence

As illustrated by the multi-dimensional nature of the study, although observing the same events, there was significant variation in the perspectives that coaches, participants and parents held. For coaching to have optimal impact and meet participant needs, there is a clear requirement to manage this range of perceptions and generate a level of coherence for all involved (Curran et al., 2021). Although the need for coherence of athlete experience is nothing new, especially in TD research (e.g., Martindale et al., 2005), social mediation of coaching remains an underexplored area in participant development. Practically, we would suggest that coaches need to build appropriate relationships with a variety of stakeholders (Jowett, 2017). These relationships can then be utilised by frequently seeking feedback from participants and parents (Nash et al., 2017).

Given the range of perceptions that may be present, effective relationships can be leveraged to deliberately shape shared mental models, enabling integration across clubs to support overall coherence (Webb et al., 2016). To achieve this, deliberate systematic communication from coach (or organisation) to participants and parents needs to be a core feature of the coaching process. It is likely that this is best done through both formal and informal means (e.g., Richards et al., 2009), with coaches reinforcing critical messages, such as the notion of challenge as a tool for development.

### Developing Perceived Competence

The longitude and multi-dimensional nature of the data demonstrates that the most prominent dilemmas for the coach appeared to be the result of biopsychosocially mediated variability within each coaching group. A prominent feature of the player experience was the mediating role of perception of competence, which appeared to have a profound impact on long-term participation, development and enjoyment of the sport (Cairney et al., 2012). This would suggest that there is a need for more nuanced thinking in youth sport (or perhaps all sporting contexts) and for coaches to see the development of perceived competence as a core feature of their practice.

The data here would clearly suggest that concepts like “just let them play” or “the game is the teacher” are inappropriate as methodologies. This is not however, to suggest that they are not appropriate methods, at the right time for the right individual. Play is obviously an important feature of participant experience, but if that play negatively impacts perceived competence and does not cater for individual differences, it is clearly a non-inclusive practice. Ultimately, mixed modalities of coaching practice that encourage perceptions of competence are likely to be important for long-term participation. This may mean that whilst skill acquisition and retention is optimally supported through desirably difficult practice (Soderstrom and Bjork, 2015), persistence and long-term motivation may not be (cf. Hodges and Lohse, 2020).

Given the need for this flexible approach to developing perceived competence and managing challenge level, in addition to preparing coaches to work within this complexity, we would suggest that sporting organisations need to offer flexibility to coaches seeking to offer variety in participation experience. For example, strategies like banning the grouping of participants by ability or mixed age groups, may be too inflexible to meet participant needs.

### Youth Sport Coaching Needs to Be Viewed Longitudinally

Despite historic characterisations of the aims and objectives of youth sport coaching being very much a short-term endeavour, with a focus on maximising short-term enjoyment, this data clearly suggests that there is a need to focus just as much on the vertical as the horizontal (Taylor and Collins, 2020). Illustrative of the complexity, it is clear that some players needed periods of carefully managed challenge-full and *less* enjoyable activity to support their long-term participation (cf. Taylor and Collins, 2019, 2021). Highlighting the complexity at the heart of the domain, this may read as contradicting the need to develop perceived competence. Instead, it highlights the need to account for individual differences, that no “one size fits all” approach can be deployed and ideally coaches need to be equipped to work in this complexity.

In addition to this, research being undertaken in the youth sport require longitude to fully explore the complexities in the coaching environment, otherwise unnoticed. The success, or otherwise, of the youth sport coaching landscape cannot be viewed on a session-by-session basis but must be considered on a variety of factors, not least the continued attendance and engagement of young people at coaching sessions over the long-term (cf. Trudel and Gilbert, 2006).

### Developing Expertise in PPW Coaching

It is often deemed acceptable for coaches working at youth sport level to have minimal experience and qualifications. This is perhaps based on long standing, short term views of the youth sport milieu. This means that many coaches working with youth PPW athletes have not developed, or been exposed to the nuanced skills of planning and adapting to their circumstances that are advocated by PJDM (Abraham et al., 2009).

Based on the experiences of coaches and participants in the present study, there is a pressing need for coaches to be prepared for the realities of the role. The current reality is that many move into youth sport coaching roles by virtue of availability, rather than expertise. This in itself clearly has the potential to lead to a poor experience of sport. Historically, many sporting organisations have offered overly simplistic guidelines, or cautious competency-based approaches. A recent trend, potentially making matters worse, has been the marketing of pre-packaged coaching solutions.

Demonstrated by these findings, there is a depth of complexity inherent in the youth coaching role, that reinforces historic calls for an expertise-based approach at all levels of coaching practice. Given the prominence of biopsychosocial factors and the role of challenge, there appears a critical need for coaches to deliberately adopt different approaches based on their intentions. An expertise-based approach facilitates this individualisation by enabling coaches to draw on a declarative knowledge base to differentiate and adapt (Nash et al., 2012).

### Greater Criticality Needs to Be Applied to the Youth Sport Coaching Environment

Finally, whilst our data set is clearly limited to one environment nested in a very specific cultural milieu, there are clear contradictions with much literature in the field of youth sport coaching. We urge that from the perspective of practice, sporting organisations take coaching practice seriously, ensuring coaches are supported appropriately, without simplistic, blanket, or procedural recommendations. Secondly, that researchers recognise the inherent complexity of any coaching environment, ensuring that theory and study design are representative of coaching reality and offer implications for real-world applied practice (Cushion et al., 2006; Collins et al., 2019).

Finally, if our data show anything, we suggest that the current body of simplistic recommendations that dominate PPW or youth sport, hold little value for the coach looking to meaningfully shape participant experience over the long-term. We may be preaching to the converted, but if we truly believe that coaching is a meaningful endeavour with the potential to shape people’s lives, then perhaps a more nuanced approach is necessary.

## Data Availability Statement

The raw data supporting the conclusions of this article will be made available by the authors, without undue reservation.

## Ethics Statement

The studies involving human participants were reviewed and approved by the Moray House School of Education, The University of Edinburgh. Written informed consent to participate in this study was provided by the participants’ legal guardian/next of kin.

## Author Contributions

CN and JT contributed to conception and design of the study and wrote the sections of the manuscript. CN carried out the fieldwork. Both authors contributed to data analysis, manuscript revision, read, and approved the submitted version.

## Conflict of Interest

JT was employed by the company Grey Matters Performance Ltd. The remaining author declares that the research was conducted in the absence of any commercial or financial relationships that could be construed as a potential conflict of interest.

## Publisher’s Note

All claims expressed in this article are solely those of the authors and do not necessarily represent those of their affiliated organizations, or those of the publisher, the editors and the reviewers. Any product that may be evaluated in this article, or claim that may be made by its manufacturer, is not guaranteed or endorsed by the publisher.
